# 2The role of controlling behaviour in intimate partner violence and its health effects: a population based study from rural Vietnam

**DOI:** 10.1186/1471-2458-9-143

**Published:** 2009-05-14

**Authors:** Gunilla Krantz, Nguyen Dang Vung

**Affiliations:** 1 Department of Community Medicine and Public Health, the Sahlgrenska Academy at the University of Gothenburg, Göteborg, Sweden; 2 Department of Demography, Faculty of Public Health, Hanoi Medical University, Hanoi, Vietnam; 3 Division of International Health, Ihcar, Department of Public Health Sciences, Karolinska Institutet, Stockholm, Sweden

## Abstract

**Background:**

Studies in North America and other high-income regions support the distinction between extreme "intimate terrorism" and occasional "situational couple violence", defined conceptually in terms of the presence or absence of controlling behaviour in the violent member of the couple. Relatively little research has been conducted on the different forms intimate partner violence may take in low-income countries. The aim of this study was to investigate whether these expressions of intimate partner violence in one low-income country, Vietnam, adhere to patterns observed in western industrialised countries as well as to investigate the resulting health effects.

**Methods:**

This cross-sectional study collected structured interview data from 883 married women aged 17–60, using the Women's Health and Life Experiences questionnaire developed by WHO. Intimate partner violence was assessed by past-year experience of physical or sexual violence and control tactics were assessed using six items combined into a scale. Three different health parameters constituted the dependent variables. Bi- and multivariate analyses, including effect modification analyses, were performed.

**Results:**

Of the participants, 81 (9.2%) had been exposed to physical or sexual violence during the past 12 months; of these, 26 (32.1%) had been subjected to one or more controlling behaviours by their partners. The risk of ill health associated with combined exposure was elevated eight to 15 times, compared to a two-fourfold risk increase after exposure to only one of the behaviours, i.e. violent acts or control tactics.

**Conclusion:**

Physical or sexual violence combined with control tactics acted synergistically to worsen health in rural Vietnamese women. The occurrence of such violence calls for altered policies, increased research and implementation of preventive and curative strategies. The unacceptability of intimate partner violence as a part of normal Vietnamese family life must be recognised in the general debate.

## Background

Most studies from low-income countries on violence in intimate relationships have investigated men's physical, mental or sexual violence against women and the related health effects. The few existing studies that include analyses on the presence or absence of controlling behaviour and the resulting health effects have mainly been conducted in the USA, Canada and the UK. The few studies from Vietnam on violence against women indicate that more than 30% of married women have been beaten or otherwise physically abused by their husbands [1-3], with serious health implications [4]. However, whether control tactics were also involved was not examined in these studies.

It has been proposed that intimate partner violence is not a single phenomenon, but consists of two distinct types of violence, defined conceptually in terms of the presence or absence of controlling behaviour in the violent member of the couple [5,6]. The critical difference between the two main types of violence, referred to as intimate terrorism and situational couple violence, is that the former is characterised by the efforts of one partner, typically the man, to systematically control the other partner, typically the woman, while situational couple violence entails unilateral or bilateral violence evolving as an escalation of conflicts into violence [5] in the absence of control tactics [6]. However, the idea of two different types of violence has been questioned in that situational couple violence may evolve into intimate terrorism via continuous escalation of the violence over time [7,8].

Situational couple violence is commonly associated with less severe injuries while the physical violence in intimate terrorism is considered to be more frequent and injurious and to become aggravated over time [5-7,9-12].

Epidemiological and clinical studies have found that physical and sexual intimate partner violence is consistently associated with a wide array of negative health outcomes, including various chronic pain syndromes, gynaecological problems, gastrointestinal disorders [13,14] and psychiatric problems, such as depression, anxiety, post-traumatic stress disorder and suicidality [15,16].

Vietnam has undergone a rapid transition during the past 20 years, moving from a planned economy system to a market economy and towards a more equal situation between men and women [17,18]. Formal equality between the sexes was established in the constitution by the socialist government in 1976. However, strong cultural traditions, often centred on patriarchal norms concerning family and traditional gender roles, continue to prevail despite their increasingly being in conflict with the economic reality men and women face [19]. Thus, men generally hold a dominant position within and outside the household while women assume primary responsibility for housework and childcare [20], although women constitute 52% of the workforce [21].

Polygamy, albeit illegal since 1960, is still practised by men in rural areas [22], and has been justified on the grounds that the family formed the main economic unit in Vietnam's traditional patriarchal society. Consequently, the more wives, daughters and female servants a man had, the more the family could produce [23]. Official documents state that polygamy is virtually non-existent today, except in some rural areas in which the law is difficult to implement [23]. However, the actual number of recognised polygamous relationships is not officially known, as they are generally not registered as marriages.

The main purpose of this analysis was to assess the prevalence of physical or sexual violence and controlling behaviours and related health effects in a representative sample of rural women in Vietnam. A further aim was to establish whether men's controlling behaviour acted synergistically with physical or sexual violence to aggravate the health outcomes. The respective characteristics and severity of violence inflicted by controlling men and non-controlling men were compared, and we presumed that any differences we might detect would support the theory of two different forms of violence.

## Methods

### Design and sample

This cross-sectional study was conducted within the framework of a demographic surveillance site, FilaBavi, in Hatay Province in northern Vietnam. FilaBavi consists of a cohort of approximately 50,000 individuals (69 clusters), selected by means of a stratified cluster-sampling procedure from the 240,000 individuals living in the district. [24] A number of households were randomly selected from each cluster, proportional to the total number of households in each cluster. The clusters were randomly selected in proportion to cluster population size, providing a self-weighted sample. Married or partnered women aged 17–60 were eligible for the study.

A structured questionnaire was employed for data collection through face-to-face interviewing. The 39 female interviewers and six field supervisors involved in the FilaBavi general data collections were trained in how to manage the specific circumstances encountered in studies of violence.

Based on power calculations, 884 households containing a married or partnered woman aged 17–60 were randomly selected for participation. Of these women, 867 were currently married and 16 were in a stable sexual relationship with a man (henceforth referred to as married women). Only one woman declined to participate due to psychiatric illness.

### Ethical considerations

The WHO guidelines for violence research were strictly followed [25]. Interviews were held in privacy, mainly in the respondents' homes, with no one able to overhear the conversation. In a few cases when privacy was not possible to establish in the home, the interview was performed at a nearby community health centre. An arrangement was made with a community health centre and the district hospital to provide treatment and support in case any of the women with experience of violence expressed the need for it. The participants were informed about the possibility to withdraw at any point during the study and they gave written informed consent to participate. Ethical approval was obtained from the Ethics Committee at the University of Gothenburg, Sweden, and from Hanoi Medical University and the Ministry of Health in Hanoi, Vietnam.

### Data collection

The Women's Health and Life Experiences Questionnaire, developed by the World Health Organisation (WHO) for violence research, was used for data collection [26]. The abuse questions were developed from other abuse assessment scales with established reliability and construct validity [11,27]. This questionnaire has been used in a number of nations in different regions of the world. Some items vary according to nation, but a core set of items are identical for all nations. This questionnaire was translated into Vietnamese and tested in pilot interviews. A review panel considered each item for appropriateness in a Vietnamese context, after which a few items were removed.

Only women participated directly in this study and data relating to husbands/partners was obtained from the participating women.

### Independent variables

*Violence prevalence *was assessed by type (physical and sexual abuse), timing (past-year exposure) and severity (moderate or severe). Physical abuse was divided into six items: slapping or throwing things and pushing or shoving, which were classified as moderate physical violence; and hitting; kicking, dragging or beating; choking or burning; and threatening or using a weapon (gun, knife or other object), which were classified as severe physical violence [11]. Sexual abuse was divided into three items: sexual intercourse against the respondent's will, using physical force to have sexual intercourse and making the respondent do something sexual that she found unnatural or degrading. Physical and sexual violence were combined as they carried the same determinants [3]; the combination is henceforth referred to as physical or sexual violence.

*Controlling behaviour *in the current husband was assessed by a composite variable made up of six items from the WHO questionnaire, presented in Table 1. The same items were used in a study by the WHO team [28] and a similar scale has also been used by Frye et al [7]. The response categories were either 'yes', assigned one point, or 'no', assigned zero points. Thus, the composite variable could yield a score ranging from 0–6 points. Internal consistency, measured by Cronbach's alpha, was .80 for the total scale. The frequency of each item and the distribution of scores in the total population are shown in Table 1. The scores were then dichotomised into controlling behaviour (≥ 1 point) as opposed to no controlling behaviour (0 points).

**Table 1:** The controlling behaviour scale.

	**Items**	**n**	**%**
1	Tries to keep her from seeing her friends		
	No	828	93.8
	Yes	55	6.2

2	Tries to restrict contact with her family of birth		
	No	855	96.8
	Yes	28	3.2

3	Insists on knowing where she is at all times		
	No	840	96.5
	Yes	31	3.5

4	Gets angry if she speaks to another man		
	No	812	92.0
	Yes	71	8.0

5	Is often suspicious that she is unfaithful		
	No	842	95.4
	Yes	41	4.6

6	Expects her to ask his permission before seeking health care for herself		
	No	861	97.5
	Yes	22	2.5

	**Composite variable scores, controlling behaviour**		
	No controlling behaviour (0 points)	769	87.1
	Controlling behaviour (1–6 points)	114	12.9

	**Controlling behaviour scores (0–6)**		

	0 point	769	87.1

	1 point	51	5.8

	2 points	28	3.2

	3 points	18	2.0

	4 points	4	0.5

	5 points	7	0.8

	6 points	6	0.7

	Total	883	100%

Socio-demographic and psychosocial variables were tested as independent risk factors. Age was divided into two groups (for respondents 17–29 and 30–60; for husbands 20–29 and 30–77). Educational level was dichotomised into primary school (≤ 5 years) and secondary and higher education (>5 years); the latter was the reference category. Annual household income was divided into quintiles and subsequently into three groups (lowest income group < USD 288, low and middle income group USD 288–570 and highest income group > USD 570) and then further dichotomised; a household income of less than USD 425 was designated as the exposure category. The respondents' occupations were dichotomised into farmers and hired labourers. Husbands' occupations were grouped into professionals, as the reference group, and semi-skilled and unskilled labourers, combined into the exposure group. Men's cohabitation status was divided into having or not having more than one recognised wife/partner.

### Dependent variables

Three health-related variables were used: women's self-reports of pain or discomfort, sadness or depression and suicidal thoughts during the 12 months preceding the survey. Pain or discomfort was dichotomised into 'no or only slight pain/discomfort' as opposed to 'moderate, severe and extreme pain/discomfort'; sadness or depression and suicidal thoughts were dichotomised into 'experience' and 'no experience' of the respective health effect.

### Statistical analysis

The Statistical Package for the Social Sciences (SPSS) version 10.0 was used for all statistical purposes [29].

Bivariate analyses estimated crude odds ratios (OR) and 95% confidence intervals for associations between socio-demographic and psychosocial variables and health effects.

To test for possible effect measure modification of controlling behaviour on the relationship between violence and the health variables, a four-category variable was constructed by combining the variables assessing the husband's controlling behaviour and past-year physical or sexual violence into dummy variables [30]. No experience of controlling behaviour or physical or sexual violence during the past 12 months constituted the reference category while all other combinations were regarded as exposure categories, i.e. being subjected to physical or sexual violence with no control tactics, or to control tactics but not to physical or sexual violence or, finally, being exposed to both. Associations between this four-category variable and the health variables were then tested in multivariate analyses, controlling for age and other statistically significant variables identified in the bivariate analyses.

## Results

### Violence prevalence and socio-demographic and psychosocial characteristics

Of the participating women, 81 (9.2%) had been exposed to physical and/or sexual violence during the past 12 months and of these, 26 (32.%) had simultaneously been subjected to one or more controlling tactics by their partner. Furthermore, 55 women were exposed to physical or sexual violence but not to control tactics and 88 women were exposed exclusively to control tactics.

The socio-demographic factors mirrored the average life circumstances in rural areas where more women than men had low-level education (21.4% and 18.0%, respectively), displayed in Table 2. Most of the husbands were semi-skilled or unskilled labourers (77%). Of the men, 15.5% (n = 130) had more than one wife/partner. Almost 12% (n = 105) of the 883 participating women reported moderate to extreme pain or discomfort, 21.9% (n = 193) suffered from sadness or depression and 3.5% (n = 31) had considered suicide during the last 12 months (Table 2).

**Table 2:** Socio-demographic and psychosocial factors and health effects N = 883.

**Variables**	**n**	**%**
***Respondents***		

**Age groups**		
17–29	198	22.4
30–60	685	77.6

**Educational level**		
Higher education	694	78.6
Primary school	189	21.4

**Occupation**		
Hired labourer (tailor, construction assistant, etc.)	122	13.8
Farmer	761	86.2

**Past-year physical or sexual violence experience**		
No	802	90.8
Yes	81	9.2

**Controlling behaviour experience from current husband**		
No	769	87.1
Yes	114	12.9

**Pain or discomfort**		
No and slight pain/discomfort	778	88.1
Moderate to extreme pain/discomfort	105	11.9

**Sadness or depression**		
No	690	78.1
Yes	193	21.9

**Suicidal thoughts**		
No	852	96.5
Yes	31	3.5

***Husbands/partners***		

**Age groups**		
20–29	108	12.5
30–77	758	87.5

**Educational level**		
Higher education	724	82.0
Primary school	159	18.0

**Socio-economic status**		
Professional	171	23.0
Semi-skilled and unskilled	573	77.0

**Has more than one wife/partner**		
No	710	84.5
Yes	130	15.5

***Households***		

**Annual income**		
< USD 288	176	20.0
USD 288–570	353	40.1
> USD 570	351	39.9

### Associations between violence, controlling behaviour and health

Bivariate associations revealed that husbands' behaviours, such as having more than one wife/partner, engaging in physical or sexual violence or control tactics, strongly influenced the respondents' health (Table 3). The women exposed to physical or sexual violence were at high risk of pain or discomfort (OR 3.75; 95% confidence interval: 2.21–6.36), of sadness or depression (OR 3.91; 2.44–6.25) and of suicidal thoughts (OR 3.07; 1.28–7.36). Control tactics were also associated with statistically significant OR for all three health variables, most strongly with suicidal thoughts (OR 4.64; 2.19–9.85). Furthermore, older age and low household income were also associated with the investigated health conditions.

**Table 3:** Associations between socio-demographic and psychosocial factors and health conditions. N = 883.

**Variables**	**Pain or discomfort**		**Sadness or depression**		**Suicidal thoughts**	
	
	**%, n**	**OR, 95%CI**	**%, n**	**OR, 95%CI**	**%, n**	**OR, 95%CI**
**Respondent's age group**						
17–29	5.6 (11)	1	15.2 (30)	1	1.5 (3)	1
30–60	13.7 (94)	2.70 (1.42–5.16)	23.8 (163)	1.75 (1.14–2.68)	4.1 (28)	2.77 (0.83–9.21)

**Respondent's education**						
Higher education	10.5 (73)	1	20.7 (144)	1	2.4 (17)	1
Primary school	16.9 (32)	1.73 (1.11–2.72)	25.9 (49)	1.34 (0.92–1.94)	7.4 (14)	3.19 (1.54–6.59)

**Husband's age group**						
20–29	9.3 (10)	1	16.7 (18)	1	2.8 (3)	1
30–77	12.0 (91)	1.34 (0.67–2.66)	22.2 (168)	1.42 (0.83–2.43)	3.6 (27)	1.29 (0.39–4.34)

**Husband's education**						
Higher education	11.5 (83)	1	21.0 (152)	1	3.2 (23)	1
Primary school	13.8 (22)	1.24 (0.75–2.06)	25.8 (41)	1.31 (0.88–1.95)	5.0 (8)	1.62 (0.71–3.68)

**Husband's SES**						
Professional	9.4 (16)	1	18.1 (31)	1	2.3 (4)	1
Semi-skilled and unskilled	10.8 (62)	1.18 (0.66–2.10)	21.6 (124)	1.25 (0.81–1.93)	3.3 (19)	1.43 (0.48–4.27)

**Household income**						
Higher income (USD 424.8–5,000)	7.9 (42)	1	16.2 (86)	1	1.9 (10)	1
Lower income (USD 0.1–424.7)	17.8 (62)	2.52 (1.66–3.82)	30.1 (105)	2.23 (1.61–3.08)	6.0 (21)	3.34 (1.55–7.17)

**Husband has > 1 wife/partner**						
No	9.4 (67)	1	18.5 (131)	1	2.8 (20)	1
Yes	24.6 (32)	3.13 (1.96–5.02)	41.5 (54)	3.14 (2.11–-4.67)	7.7 (10)	2.88 (1.31–6.29)

**Respondents' experience of past-year physical or sexual violence**						
No	10.1 (81)	1	19.2 (154)	1	3.0 (24)	1
Yes	29.6 (24)	3.75 (2.21–6.36)	48.1 (39)	3.91 (2.44–6.25)	8.6 (7)	3.07 (1.28–7.36)

**Respondents' experience of controlling behaviour**						
No (0 point)	9.6 (74)	1	19.8 (152)	1	2.5 (19)	1
Yes (1–6 points)	27.2 (31)	3.51 (2.18–5.65)	36.0 (41)	2.28 (1.50–3.48)	10.5 (12)	4.64 (2.19–9.85)

As one of the main purposes of this study was to investigate whether simultaneous exposure to physical or sexual violence and control tactics would aggravate health outcome, effect measure modification analyses were performed with the four-category variable. In Table 4, crude and adjusted odds ratios are presented for the three health conditions.

**Table 4:** Interaction effect of controlling behaviour on the association between past-year physical or sexual violence and health conditions.

**Variables**	**Pain or discomfort**		**Sadness or depression**		**Suicidal thoughts**	
	
	**COR** **95%CI**	AOR * 95%CI	**COR** **95%CI**	AOR** 95%CI	**COR** **95%CI**	AOR*** 95%CI
No controlling behaviour and no past-year phy/sex violence **(n = 714)**	1	1	1	1	1	1

Controlling behaviour but no past-year phy/sex violence **(n = 88)**	2.43 (1.35–4.38)	2.04 (1.09–3.83)	1.80 (1.09–2.97)	1.51 (0.89–2.58)	3.54 (1.43–8.80)	3.06 (1.19–7.86)

No controlling behaviour but subjected to past-year phy/sex violence **(n = 55)**	2.26 (1.09–4.69)	2.44 (1.14–5.25)	3.26 (1.85–5.76)	3.60 (1.98–6.55)	1.55 (0.35–6.88)	1.40 (0.31–6.35)

Controlling behaviour and subjected to past-year phy/sex violence **(n = 26)**	11.85 (5.26–26.71)	15.37 (6.22–37.94)	7.26 (3.22–16.36)	8.58 (3.57–20.66)	9.76 (3.29–28.96)	10.80 (3.42–34.11)

Crude (COR) and adjusted (AOR) odds ratios. N = 883

* adjusted for respondents' age, respondents' education, household income and husband having > 1 wife/partner

** adjusted for respondents' age, household income and husband having > 1 wife/partner

*** adjusted for respondent's age, respondents' education, household income and husband having > 1 wife/partner

Twenty-six women suffered physical or sexual violence including control tactics. Such combined exposure increased the associations with ill health considerably, i.e. OR 15.4 (6.2–37.9) for pain/discomfort, OR 8.6 (3.6–20.7) for sadness/depression and OR 10.8 (3.4–34.1) for suicidal thoughts, after adjustment for the statistically significant variables detected in the bivariate analyses (respondents' age, respondents' education, household income and husband having > 1 wife/partner). Experience of violence was a more serious threat to women's health than controlling behaviour with one exception. For the 31 respondents reporting suicidal thoughts, exposure to control tactics was the stronger risk factor (OR 3.06; 1.19–7.86).

Analyses to compare controlling and non-controlling men revealed that the controlling men were younger and less well-educated, lived in poorer households and more often had multiple wives/partners than the non-controlling men.

The assumption that violence inflicted through combined exposure would be more serious than violence inflicted without any control tactics was confirmed. We found that 19 (73%) of the 26 women subjected to violence and control tactics had experienced severe physical violence while the majority of the women exposed to physical violence alone were victims of moderate physical violence.

## Discussion

Physical or sexual violence perpetrated by husbands is fairly common in rural Vietnam. Nine percent of the total population of women had been exposed during the past year and almost 13 percent had endured control tactics. For the women who reported combined exposure, the risk of ill-health was elevated eight to 15 times, compared to a two-fourfold risk increase in ill health when exposed to only one of the behaviours, illustrating how violent acts and control tactics acted synergistically to impair health.

We further found that perpetrators of violence who also used control tactics differed somewhat in their characteristics, such as being younger, and more often had multiple recognised partners, compared to men who did not use control tactics. An additional finding was that violence classified as 'severe' dominated in cases of combined exposure, while 'moderate' violence occurred more often in cases of physical or sexual violence alone.

### Methodological considerations

In this study, we examined past-year occurrence of physical and/or sexual violence as well as three different health conditions during the same time period. Past-year occurrence of violence is often thought to be a more accurate assessment of intimate partner violence because of the assumption of less recall bias. As women in general, including in Vietnam, are not immediately willing to disclose violence experience, there is a risk of under-reporting. However, the data collection procedure in this study was performed with great care by experienced female interviewers and closely supervised and the data is considered to be of high quality. The possibility that women reporting coercion but no physical violence might have underreported violence incidents cannot be ruled out.

One limitation of this study is that the woman reported the husband's behaviours, i.e. the use of violence and control tactics, as this might have resulted in exaggeration. On the other hand, this ensures that male controlling behaviour and violence perpetration is reported at all, as men might hesitate to report their own offences. This procedure has been used by other researchers and is considered to reflect the actual situation fairly accurately [31].

The controlling behaviour index was made up of six control tactic items with a cut-off point at 1. As the inter-item correlation of our scale was high, there was no reason to delete any of the items. It could be argued that the criteria for controlling behaviour should have been stricter. However, the six included items mirrored serious attempts to control time, contacts or access to health care, and no single item could be regarded as acceptable relationship behaviour. Moreover, some partners might employ just one tactic; it might well be the frequency rather than the number of tactics that makes a difference. This enables the conclusion that the methodology and instrument used were adequate for this study.

Health conditions such as pain/discomfort and sadness/depression are fairly common in the general population and might reflect a state of stress [32], while suicidal thoughts contitute a more precise and serious health outcome. However, strong potential confounding factors were controlled for. Most of them proved to be independently associated with the chosen health conditions and the crude estimates only changed to a limited extent. This supports our notion that these health variables adequately reflect the effects of such violations against women.

As with all cross-sectional studies, it is not possible to establish the direction of associations. It is, however, well known from longitudinal studies that serious health conditions result from exposure to violence [33].

The findings in this study must be followed up by further studies, since the sample size of some of our sub-populations was small due to the study being population-based and not conducted among a population of women subjected to physical violence. There is a need also for qualitative studies to explore individual women's personal experience of violence combined with control tactics.

### Our results in relation to other studies

Violence and abuse indisputably result in diverse and serious health effects in women [10,15,32,34], but rather few studies have as yet investigated the combined effect of violence and controlling behaviour. However, in support of our findings, Leone found, in a study from Chicago, that victims of intimate terrorism reported poorer general health, a greater likelihood of visiting a doctor and more symptoms of post-traumatic stress disorder than those subjected to physical violence alone [10].

Our finding that controlling behaviour was related to a higher risk of suicidal thoughts than actual physical or sexual violence requires comment as it contrasts with findings concerning the other two health variables. One possible explanation is that being exposed to violence is part of everyday life for many Vietnamese women [20,35,36], while controlling behaviour is an invisible violation, difficult to disclose, that may be psychologically more detrimental, consequently leading to more serious health effects.

Our results also show that individual characteristics and attitudes among the controlling men differed from those who were not controlling. The controlling men were younger and more often had more than one recognised partner, they were also poorer and mainly had low-level education but these latter characteristics also pertain to men who inflict physical violence [1,3,37]. So the important question is whether particular types of men engage exclusively in controlling behaviours or whether this is just part of the previously described violence escalation pattern, starting with milder forms of psychological abuse but escalating over time into controlling behaviour and later into serious forms of physical violence [8]. Our findings give some support to the hypothesis that violence with and without controlling behaviour occur as separate entities [5,6] but a longitudinal study is required to adequately discriminate between the two types.

## Conclusion

Two thirds of the cases of physical or sexual violence were not combined with control tactics. The respective characteristics of the men who engaged in physical or sexual violence with and without controlling behaviours differed and the violence inflicted by controlling men was more severe in nature than that inflicted by non-controlling men. A reflection of the latter finding was that women's risk of ill-health increased many times when exposed to both violations.

These results, presented for the first time from a low-income country, are in line with findings from this type of investigation in a few high-income countries. The findings underscore the idea that mechanisms behind violence perpetration may be universal and points at the assumption that different types of violence co-exist in populations. However, these findings must be investigated in more detail, among other reasons because the sample size in some of our sub-populations was small.

The extremely high health risks associated with partner violence combined with control tactics call for altered policies and legal action against perpetrators, increased research and implementation of preventive and curative strategies. The unacceptability of intimate partner violence as a part of modern family life should be recognised in the general debate and presented as a serious violation of gender equity ideals. Legal and social support mechanisms for abused women and their children needs to be established at the local level and health care staff should be trained in the professional consultation and care of violence victims. The social ideal of gender equity, stipulated in official documents, must be implemented in all sectors of society.

## Competing interests

The authors declare that they have no competing interests.

## Authors' contributions

NDV was responsible for data collection, performed all the analyses and drafted a first version of the manuscript. GK planned the study, further contributed to data collection and decided which analyses to perform. GK made all revisions of the manuscript before submission. Both authors had full access to all data and the corresponding author made the final decision to submit this version.

## Pre-publication history

The pre-publication history for this paper can be accessed here:


